# ASO Author Reflections: Employment Disruption and Financial Burden Associated with Gastrointestinal Cancers

**DOI:** 10.1245/s10434-025-17734-7

**Published:** 2025-06-21

**Authors:** Mujtaba Khalil, Timothy M. Pawlik

**Affiliations:** Department of Surgery, The Urban Meyer III and Shelley Meyer Chair for Cancer Research, The Ohio State University, Columbus, USA

## Past

Cancer treatment is complex and often involves a combination of surgery, radiation, and chemotherapy.^1^ Surgery is inherently invasive, and both chemotherapy and radiation therapy can lead to significant systemic toxicities.^2^ As a result, patients undergoing cancer treatment experience physical challenges such as pain, fatigue, and digestive issues, which severely impact their quality of life.^3^ Moreover, the emotional toll of battling cancer can result in mental health issues such as depression and anxiety.^3^ The financial burden including high medical costs and potential loss of income due to an inability to work further exacerbates these challenges for both patients and their families.^4^ As such, understanding the risk factors for employment disruption can help inform strategies to facilitate a return to work. The current study sought to characterize employment patterns and financial challenges in the year following gastrointestinal (GI) cancer diagnosis.^5^

## Present

A total of 11,832 individuals with GI cancer were included in the analytic cohort. Median patient age was 54 years (IQR 49–59 years) with a majority of patients being male (*n* = 7054; 59.6%). In the year following GI cancer diagnosis, 13.8% (*n* = 1638) of patients experienced employment disruption. Notably, individuals working in finance (HR 1.66, 95% CI 1.37–2.02), manufacturing (HR 1.19, 95% CI 1.01–1.42), and the transportation industry (HR 1.33, 95% CI 1.11–1.60) were at a higher risk of experiencing employment disruption. Patients with GI cancer had higher out-of-pocket costs ($3250 [IQR $1756–5255] versus $453 [IQR $142–1189]), more workdays missed (50 days [IQR 28–72 days] versus 5 days [IQR 2–10 days]) (both *p* < 0.001). Overall, GI cancer diagnosis was associated with threefold higher risk of employment disruption (HR 3.16, 95% CI 2.96–3.38).

## Future

Recent advancements in cancer care have led to improved long-term outcomes.^6^ However, patients continue to experience considerable personal, social, and financial challenges during their treatment journey.^3^ Notably, the risk of employment disruption increased threefold during the first year following GI cancer diagnosis.^5^ Additionally, patients with GI cancer experienced more missed workdays and greater financial burdens.^5^ Addressing these challenges requires a coordinated approach involving healthcare providers, employers, and policymakers.^7,8^ Healthcare teams should discuss potential employment challenges with patients early in treatment, helping them plan for workplace accommodations and leave options.^7^ Employers can support patients by offering flexible work arrangements, paid sick leave, and modified job duties, while comprehensive return-to-work programs that include physical, psychosocial, and educational components can facilitate reintegration into the workplace.^7^ At the policy level, expanding leave policies and improving access to temporary disability insurance can better protect patients against lost wages and job loss.^8^ Developing financial navigation services and screening tools to identify patients at risk of financial hardship can also help connect them with appropriate resources.^4^ By addressing these factors collectively, the economic impact on patients with cancer and their families can be mitigated, ultimately improving their quality of life and treatment outcomes.^4,8^
